# Challenges and opportunities for engineering thermochemistry in carbon-neutralization technologies

**DOI:** 10.1093/nsr/nwac217

**Published:** 2022-10-14

**Authors:** Guangwen Xu, Dingrong Bai, Chunming Xu, Mingyuan He

**Affiliations:** Key Laboratory on Resources Chemicals and Materials, Shenyang University of Chemical Technology, China; Key Laboratory on Resources Chemicals and Materials, Shenyang University of Chemical Technology, China; College of Chemical Engineering and Environment, China University of Petroleum-Beijing, China; Shanghai Key Laboratory of Green Chemistry & Chemical Processes, Department of Chemistry, East China Normal University, China

## Abstract

Engineering thermochemistry is the science and technology that studies, innovates, and engineers heat-induced or heat-driven thermochemical reactions and can potentially lead to reductions of five-plus billion tons of CO_2_ emissions effectively and economically.

Stalling and neutralizing carbon emissions while maintaining the quality of life is a global challenge of our time. With >80% of the world's energy and materials (e.g. steel, cement, plastics) from fossil and carbonaceous resources, achieving this goal in a short time needs scientifically sound, economically sustainable and practically effective and deployable approaches, even though many strategies have been proposed by researchers and policymakers [1,2]. Statistical analysis indicates that excessive carbon emissions are from several super-emitters, including boilers and motors burning fossil fuels (i.e. coal, oil and natural gas) for the generation of power or driving force, industrial combustion for heat and steam generation, electrolytic and electric-arc furnaces for ultra-high-temperature mineral-processing or metallurgical processes (calcium carbide, silicon, fused magnesia, etc.) and high-temperature kilns for the production of cement, lime and refractory materials (Fig. 1). These super-emitters emit >90% of China's ∼10 billion tons of CO_2_ emissions [3]. Reducing CO_2_ from these super-emitters effectively curbs and neutralizes carbon emissions and must be the top priority.

**Figure 1.**
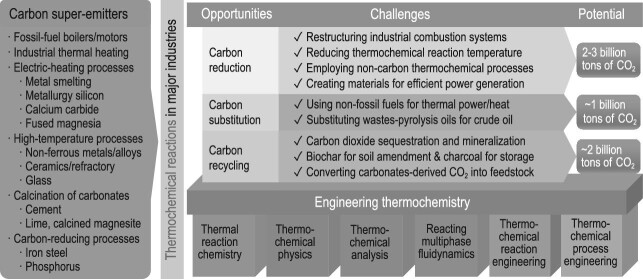
Opportunities, challenges and potential for engineering thermochemistry toward carbon neutralization.

We have realized that almost all of the aforementioned CO_2_ super-emitters involve heat-induced or heat-driven thermochemical reactions, such as combustion, gasification, decomposition, calcination, reduction and sintering [4].

These reactions are wildly popular in diversified industries, including energy, transportation, chemicals, metallurgy, mineral processing and the environment, to produce electricity, heat, power, materials and other products essential to our life. Innovating and revolutionizing these thermochemical reaction processes are critical to carbon neutrality, but there are many challenges because existing sciences and engineering have never imposed the demand for carbon reduction on a scale of billions of tons.

Engineering thermochemistry (ETC), established by Chinese scientists [5], is the science and technology concerning heat-induced/driven thermochemical reactions and their engineering. It studies heat-induced/heat-driven thermochemical phenomena and reaction behavior of substances in various atmospheres; innovates the thermochemical reaction process, reactor and system to transform the substances into value-added products safely, efficiently and economically; and integrates and harmonizes the fundamentals and engineering aspects of thermochemical reactions, which used to be dealt with separately in various scientific disciplines and industries. Treating these reactions with a more systematic and disciplined approach, ETC can lead to scientific breakthroughs and technological innovations toward carbon neutrality through the pathways of reduction, substitution and recycling of carbon (Fig. 1).

Reducing carbon emissions must chase after CO_2_’s super-emitters and we have identified the following opportunities and approaches. (i) Increasing efficiency of heating and power generation. Of the carbonaceous fuels consumed in China, >60% are directly or indirectly combusted to generate power, steam or heat. China's district/industrial heating furnaces have thermal efficiencies ≥20 percentage points lower than the advanced international standards. The power generation based on thermal energy is limited by the Carnot cycle, which delivers ∼40% in fuel-to-electricity efficiency. In terms of reducing carbon emissions, the use of carbon-based fuels, especially coal, should ideally be reduced drastically or phased out altogether. However, this will not happen anytime soon, given energy source availability and technical reality. Nevertheless, it is still possible to reduce billions of tons of CO_2_ emissions from these carbon-intensive processes by, for example, restructuring industrial combustion reactions to lower O_2_/air consumption and creating advanced high-temperature engineering materials (e.g. metal alloys withstanding well over 700ºC for ultra-supercritical combined cycles, complex materials used in thermoelectric power generation at temperatures of >1000ºC) to realize high-efficiency power generation. (ii) Switching electric heating to thermal energy for high-temperature processes. Many essential materials (e.g. Fe, Cu, Al, Cu, Si, CaC_2_ and fused magnesia) are produced industrially by electrolytic or electric-heating reactors. These processes, consuming >10% of China's electricity, have a primary energy efficiency of <20%. Instead of using electricity, using thermal energy directly in these thermochemical technologies, which can be invented by following the ETC principles, will increase the energy efficiency of such energy-intensive processes to >80%. (iii) Lowering process temperature through process intensification with small-size feedstock. Chunk feedstocks are generally used to facilitate commercial operations of shaft or rotary kilns in manufacturing cement, lime, glass, pig iron and many other materials. These processes feature high temperatures (>2000°C) and long reaction times because large-size feedstocks increase the heat-transfer resistance drastically. In the principle of ETC, processing small particles in fluidized beds can effectively remove the heat-transfer barrier and allow reactions to complete at significantly reduced temperatures and times, thus contributing considerably to energy and CO_2_ reductions [6,7]. (iv) Developing non-carbon thermochemical processes. Carbon has been essential to many industries. It has been used as a reductant in manufacturing many materials (e.g. Fe, Mg and Si), as electrodes in electric-arc furnaces and as additives in many products (e.g. tires, steels). Thus, a significant reduction in CO_2_ emissions can be achieved by breakthrough technologies in which fossil carbon can be replaced by non-carbon or less-carbon reagents. The direct reduction of iron [8] and decomposition of carbonates (e.g. cement production) [9] using H_2_-rich gas (especially green hydrogen) are perfect examples of such actively pursuing attempts, in which many issues in relation to thermal, reaction and engineering problems can be solved by approaches of ETC.

Substituting the carbon of renewable and recyclable sources for fossil carbon is a sustainable route to carbon neutrality while enabling the continuous manufacturing of irreplaceable carbonaceous materials and the production of heat/electricity. ETC plays a critical role in making this possible by developing advanced thermochemical processes and reactors to produce energy and non-fossil carbonaceous feedstock. For example, the petroleum industry can use alternative hydrocarbon fuels, including biomass, biogas and organic debris (e.g. plastic wastes), to generate liquid, volatiles and char feedstocks to substitute for crudes, natural gas and fossil carbon, respectively [10]. In fact, carbon substitution alone can potentially reduce China's carbon footprint by up to one billion tons annually.

Carbon recycling ultimately leads to establishing a carbon-neutral circular economy. ETC provides approaches to mimic underground sequestration or mineralization of CO_2_ in a thermochemical reactor but with significantly accelerated rates [11]. ETC also directs the development and deployment of conversion technologies, catalytically or non-catalytically, to convert CO_2_ from various sources into value-added products. With advanced thermochemical reaction technologies, ∼700 million tons of high-purity CO_2_ released from processing various carbonates (e.g. cement, lime) can be further purified and converted into carbonaceous feedstocks and chemicals. Reductive decompositions of carbonates have a broad application prospect because they produce valuable materials and syngas while reducing CO_2_ emissions and improving the process economics [9,12]. Pyrolysing carbonaceous fuels can produce gas and liquid products for beneficial uses and char for soil amendment or storage. In China, all these measures of carbon recycling allow ∼2 billion tons of carbondioxide reduction [13].

Overall, ETC provides great opportunities for revolutionizing thermochemical reaction processes, especially those carbon super-emitters, and has the potential to result in >5 billion tons of CO_2_ emissions reduction in China. Doubtlessly, neutralizing carbon emissions will eventually require replacing fossil resources with green and renewable resources (such as solar, wind, hydropower and geothermal). On the road to this destination, ETC will play a vital role in transforming, developing and integrating new energy systems, including solar energy, green electricity and hydrogen. To realize the ETC’s full potential, we have addressed the scientific, technological and implementation challenges in this short paper.
